# Functional Characterization of Two Class II Diterpene Synthases Indicates Additional Specialized Diterpenoid Pathways in Maize (*Zea mays*)

**DOI:** 10.3389/fpls.2018.01542

**Published:** 2018-10-23

**Authors:** Katherine M. Murphy, Li-Ting Ma, Yezhang Ding, Eric A. Schmelz, Philipp Zerbe

**Affiliations:** ^1^Department of Plant Biology, University of California, Davis, Davis, CA, United States; ^2^School of Forestry and Resource Conservation, National Taiwan University, Taipei, Taiwan; ^3^Section of Cell and Developmental Biology, University of California, San Diego, La Jolla, CA, United States

**Keywords:** diterpene synthase, diterpenoid biosynthesis, plant stress response, plant specialized metabolism, *Zea mays*

## Abstract

As a major staple food, maize (*Zea mays*) is critical to food security. Shifting environmental pressures increasingly hamper crop defense capacities, causing expanded harvest loss. Specialized labdane-type diterpenoids are key components of maize chemical defense and ecological adaptation. Labdane diterpenoid biosynthesis most commonly requires the pairwise activity of class II and class I diterpene synthases (diTPSs) that convert the central precursor geranylgeranyl diphosphate into distinct diterpenoid scaffolds. Two maize class II diTPSs, ANTHER EAR 1 and 2 (ZmAN1/2), have been previously identified as catalytically redundant *ent*-copalyl diphosphate (CPP) synthases. ZmAN1 is essential for gibberellin phytohormone biosynthesis, whereas ZmAN2 is stress-inducible and governs the formation of defensive kauralexin and dolabralexin diterpenoids. Here, we report the biochemical characterization of the two remaining class II diTPSs present in the maize genome, COPALYL DIPHOSPHATE SYNTHASE 3 (ZmCPS3) and COPALYL DIPHOSPHATE SYNTHASE 4 (ZmCPS4). Functional analysis via microbial co-expression assays identified ZmCPS3 as a (+)-CPP synthase, with functionally conserved orthologs occurring in wheat (*Triticum aestivum*) and numerous dicot species. ZmCPS4 formed the unusual prenyl diphosphate, 8,13-CPP (labda-8,13-dien-15-yl diphosphate), as verified by mass spectrometry and nuclear magnetic resonance. As a minor product, ZmCPS4 also produced labda-13-en-8-ol diphosphate (LPP). Root gene expression profiles did not indicate an inducible role of *ZmCPS3* in maize stress responses. By contrast, *ZmCPS4* showed a pattern of inducible gene expression in roots exposed to oxidative stress, supporting a possible role in abiotic stress responses. Identification of the catalytic activities of ZmCPS3 and ZmCPS4 clarifies the first committed reactions controlling the diversity of defensive diterpenoids in maize, and suggests the existence of additional yet undiscovered diterpenoid pathways.

## Introduction

Plant labdane-related diterpenoids represent a diverse group of more than 7,000 metabolites with broad physiological functions in plant development, defense, and ecological adaptation (Peters, 2010; Zerbe and Bohlmann, 2015). A few widely conserved diterpenoid metabolites and the corresponding metabolic enzymes are essential for gibberellin biosynthesis of general metabolism (Richards et al., 2001; Zi et al., 2014). From these ancestral diterpenoid pathways, the vast chemical space of specialized diterpenoids has evolved (Zi et al., 2014), which comprises common and species-specific diterpenoid blends with distinct biological functions that range from pest and pathogen defense to allelopathic and signaling activities (Keeling and Bohlmann, 2006a; Chaturvedi et al., 2012; Schmelz et al., 2014; Tholl, 2015).

**FIGURE 1:**
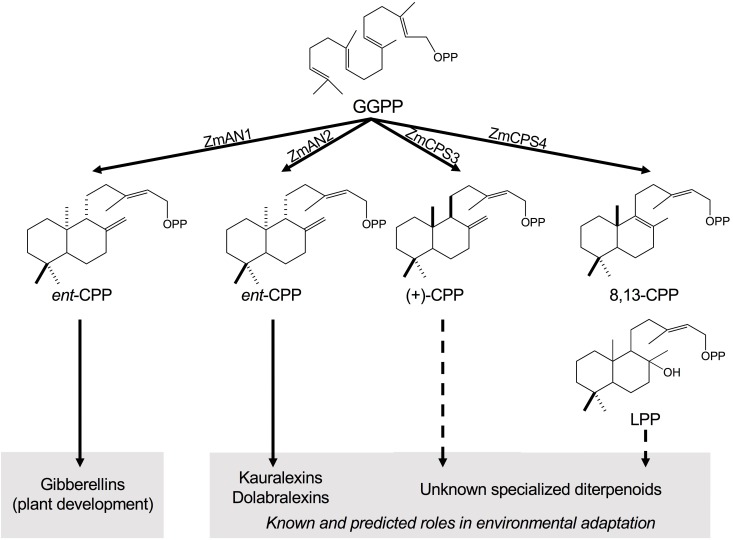
Class II diterpene synthase functions in maize diterpenoid metabolism. Maize deploys functionally diverse families of class II and class I diterpene synthases (diTPSs) that act sequentially to form the core scaffolds of diverse biologically active diterpenoids that mediate plant growth and responses to both biotic and abiotic stress. Among the four class II diTPSs present in the maize genome, previous studies showed that ZmAN1 functions in gibberellin phytohormone biosynthesis, whereas ZmAN2 controls the formation of kauralexin and dolabralexin diterpenoids as major components of the maize chemical defense system (Schmelz et al., 2011; Mafu et al., 2018). ZmCPS3 and ZmCPS4 represent additional specialized class II diTPSs that form (+)-CPP and 8,13-CPP as major products, respectively, representing previously hidden precursors of maize specialized diterpenoid metabolism. GGPP, geranylgeranyl diphosphate; CPP, copalyl diphosphate; LPP, labda-13-en-8-ol diphosphate.

Two of the most agroeconomically important grain crops, maize (*Zea mays*) and rice (*Oryza sativa*), deploy distinct networks of diterpenoids to mediate the plant response to biotic and abiotic stress (for review, see Peters, 2006; Schmelz et al., 2014). Rice produces a complex suite of diterpenoid phytoalexins, including momilactones, oryzalexins, and phytocassanes, that serve as major components of disease resistance and exhibit allelopathic properties to suppress the growth of competing weeds (Peters, 2006; Kato-Noguchi and Peters, 2013; Toyomasu et al., 2014; Lu et al., 2018). Similarly, maize produces related yet distinct arsenals of bioactive diterpenoids that, to current knowledge, include the kauralexin and dolabralexin groups with demonstrated and predicted functions in maize chemical defense (Figure 1; Schmelz et al., 2011; Vaughan et al., 2015; Christensen et al., 2018; Mafu et al., 2018). For example, pathogen-elicited maize kauralexin and dolabralexin diterpenoids exhibit potent antimicrobial efficacies *in vitro* and *in vivo* against several pathogens (Schmelz et al., 2011; Christensen et al., 2018; Mafu et al., 2018), including species of *Fusarium* as a major causal agent of maize crop losses and mycotoxin contamination (Mueller, 2012). In addition, kauralexins exhibit antifeedant activity in response to herbivore attack by the European corn borer (*Ostrinia nubilalis*; Dafoe et al., 2011; Schmelz et al., 2011). More recently, both kauralexins and dolabralexins have been shown to accumulate under drought and below-ground oxidative stress, consistent with a possible protective role of diterpenoids in abiotic stress responses (Vaughan et al., 2015; Mafu et al., 2018). Furthermore, a kauralexin- and dolabralexin-deficient maize mutant *an2* (*anther ear 2*) showed increased susceptibility to both drought stress and pathogen attack (Vaughan et al., 2015; Christensen et al., 2018). These findings highlight the biological importance of diterpenoids in conferring stress resilience, and underscore the need to better understand the biosynthesis and chemical ecology of diterpenoids in cereal crops.

In angiosperms, the structural diversity of labdane-type diterpenoids is determined by the activity of class II and class I diTPS enzymes that act sequentially to transform the central precursor GGPP into different scaffolds (Peters, 2010; Zerbe et al., 2015). Class II diTPSs control the first committed reaction, catalyzing the protonation-initiated cyclization of GGPP into bicyclic labdadienyl/CPP intermediates of distinct stereochemistry and/or regio-specific oxygenations (Peters, 2010; Zerbe and Bohlmann, 2015). Class I diTPSs then convert these intermediates via ionization of the diphosphate group and a variety of possible downstream cyclization and rearrangement reactions of the intermediary carbocation (Peters, 2010; Zerbe and Bohlmann, 2015). Multi-gene diTPS families of 9–31 members were identified in the genomes of maize, rice, wheat (*Triticum aestivum*), and switchgrass (*Panicum virgatum*; Harris et al., 2005; Xu et al., 2007a; Wu et al., 2012; Zhou et al., 2012; Mafu et al., 2018; Pelot et al., 2018). Functional characterization of class II diTPSs in these species demonstrated the formation of both common and distinct CPP stereoisomers. All species produce *ent*-CPP as a precursor for gibberellin phytohormones, as well as specialized diterpenoids at least in rice and maize (Schmelz et al., 2014). In addition, wheat forms the enantiomeric (+)-CPP en route to pimarane and abietane diterpenoids, whereas rice and switchgrass form *syn*-CPP en route to, for example, rice oryzalexins and momilactones (Xu et al., 2004, 2007a; Wu et al., 2012; Zhou et al., 2012; Pelot et al., 2018). The switchgrass class II diTPS family appears to have functionally diverged more extensively to also produce 8,13-CPP (labda-8,13-dien-15-yl diphosphate) and the clerodane diterpenoid precursor clerodienyl diphosphate (Pelot et al., 2018). In maize, only two of the four class II diTPSs present in the genome (B73 RefGen_v4), namely, ANTHER EAR 1 (ZmAN1, Zm00001d032961) and ANTHER EAR 2 (ZmAN2, Zm00001d029648), have been functionally analyzed and demonstrated to produce *ent*-CPP (Bensen et al., 1995; Harris et al., 2005). Despite their catalytic redundancy, ZmAN1 and ZmAN2 serve different physiological functions in maize. Knock-out mutants of *Zman1* display characteristic gibberellin-deficient phenotypes including dwarfism and anther formation in ears that demonstrate a role of ZmAN1 in general metabolism (Bensen et al., 1995). By contrast, ZmAN2 is stress-inducible and *an2* mutants exhibit normal growth and reproductive phenotypes, but display enhanced susceptibility to fungal disease and environmental stress associated with the lack of kauralexins and/or dolabralexins (Harris et al., 2005; Vaughan et al., 2015; Christensen et al., 2018; Mafu et al., 2018). While these genetic studies clarified the dependency of kauralexin and dolabralexin metabolism on ZmAN2 activity, knowledge of the downstream class I diTPS reactions remains incomplete. Only KAURENE SYNTHASE-LIKE 4 (ZmKSL4, Zm00001d032858) and the P450s CYP71Z16/18 (Zm00001d014136/Zm00001d014134) have been recently identified to catalyze the conversion of *ent*-CPP into dolabradiene and downstream dolabralexins (Mafu et al., 2018). These enzymes expand our knowledge of previously reported maize *ent*-kaurene synthases (ZmKSL3, ZmKSL5, and ZmTPS1) that convert *ent*-CPP into *ent*-kaurene en route to gibberellin biosynthesis and possibly specialized diterpenoid metabolism (Fu et al., 2016).

Herein reported is the biochemical characterization of the two remaining class II diTPSs present in the maize genome, COPALYL DIPHOSPHATE SYNTHASE 3 (ZmCPS3, Zm00001d024512) and COPALYL DIPHOSPHATE SYNTHASE 4 (ZmCPS4, Zm00001d048874), to delineate the scope of yet unknown specialized diterpenoid pathways in maize. Unlike ZmAN2, interrogation of transcriptomic and proteomic datasets did not support a role of ZmCPS3 and ZmCPS4 in highly inducible pathogen defenses. A pattern of largely constitutive gene expression of *ZmCPS3* and moderately inducible expression of *ZmCPS4* under root exposure to abiotic stress may suggest more constitutive functions in maize ecological adaptation.

## Materials and Methods

### Gene Constructs

Full-length genes of maize *ZmCPS3* (Zm00001d024512) and *ZmCPS4* (Zm00001d048874) were synthesized with support of a Department of Energy Joint Genome Institute Community Science Program grant (CSP#2568). Genes were inserted into the second multiple cloning site of a pACYC-Duet plasmid also carrying the GGPP synthase from *Abies grandis* (Morrone et al., 2010) to form the constructs pACYC-Duet:AgGGPPS-ZmCPS3 and pACYC-Duet:AgGGPPS-ZmCPS4 (Supplementary Table S1). Additional constructs used in co-expression assays were described previously, including pACYC-Duet:AgGGPPS-ZmAN2 (Zm00001d029648) (*Z. mays ent*-CPP synthase), pET28b:ZmKSL3 (Zm00001d002349) (*Z. mays ent*-kaurene synthase), pET28b:ZmKSL4 (Zm00001d032858) (*Z. mays* dolabradiene synthase), pET28b:GrTPS1 (*Grindelia robusta* LPP synthase), and pET15b:MvELS (*Marrubium vulgare* 9,13-epoxy labd-14-ene synthase; Zerbe et al., 2013, 2014; Mafu et al., 2018).

### Enzyme Functional Analysis

Functional co-expression of enzymes was carried out using an engineered *Escherichia coli* platform for enhanced diterpenoid production (Morrone et al., 2010) as previously described (Mafu et al., 2018). The pACYC-Duet:AgGGPPS-ZmCPS3, pACYC-Duet:AgGGPPS-ZmCPS4, or pACYC-Duet:AgGGPPS-ZmAN2 constructs were expressed alone or in combination with ZmKSL3, or ZmKSL4. GrTPS1 was expressed in combination with pACYC-Duet:AgGGPPS to form LPP and an additional combination with pET15b:MvELS to form manoyl oxide for use as authentic standards. In brief, cultures were grown in 50 mL Terrific Broth (TB) medium to an OD_600_ of ∼0.6 at 37°C. After cooling to 16°C, cultures were induced with 1 mM isopropyl-thio-galactoside (IPTG) and 25 mM sodium pyruvate and incubated for a further 72 h. Enzyme products were extracted with 50 mL of hexane, concentrated under air, and resuspended in 1 mL hexane for GC-MS analysis.

### GC-MS Analysis

Gas chromatography mass spectrometry analysis of enzyme products was performed on an Agilent 7890B GC interfaced with a 5977 Extractor XL MS Detector at 70 eV and 1.2 mL min^-1^ He flow, using an Agilent HP5-MS column (30 m, 250 μm i.d., 0.25 μm film) with a sample volume of 1 μL and the following GC parameters: pulsed splitless injection at 250 and 50°C oven temperature; hold at 50°C for 3 min, 20°C min^-1^ to 300°C, hold 3 min. MS data from 90 to 600 mass-to-charge ratio (*m*/*z*) were collected after a 8 min solvent delay. Products were identified using comparison to authentic standards or, where these were not available, comparison to published mass spectra and the National Institute of Standards and Technology (NIST version 2.0) mass spectral library (Agilent).

### NMR Analysis

Diterpenoids were produced via large-scale (12 L) enzyme co-expression cultures as described above. Hexane extracts were dried using rotary evaporation, resuspended in hexane, and purified by silica column chromatography (230–400 mesh, grade 60) using a hexane:ethyl acetate gradient as the mobile phase. Fractions were further purified on an Agilent 1100 series HPLC with diode array UV detector and an Agilent ZORBAX Eclipse Plus-C8 column (4.6 mm × 150 mm, 5 microns) at a 0.5 mL min^-1^ flow rate and H_2_O/acetonitrile gradient as mobile phase. Product purity was verified using GC-MS analysis as outlined above. Purified products were dissolved in 0.6 mL deuterated chloroform (CDCl_3_; Sigma-Aldrich) containing tetramethylsilane (TMS). NMR spectra were acquired at room temperature on a Bruker Avance III 800 spectrometer equipped with a 5 mm CPTCI. Chemical shifts were calculated by reference to known CDCl_3_ (^13^C 77.23 ppm, ^1^H 7.24 ppm) signals offset from TMS. All spectra were acquired using standard experiments on a Bruker TopSpin 3.2 software, including 1D ^1^H and 1D ^13^C spectra (201 MHz).

### Transcriptomics and Proteomics Analysis

For analysis of class II diTPS gene and protein abundance, publicly available transcriptome and proteome inventories were investigated that represent a range of organs and tissues at different developmental stages of healthy maize plants (Walley et al., 2016). All samples derive from B73, with the exception of 2 cm tassels, 1–2 mm anthers, and mature pollen (W23 inbred), and 5-days-old primary root (Mo17 inbred) (Walley et al., 2016). Transcript abundance (as fragments per kilobase of transcript per million mapped reads, FPKM) and protein expression levels were retrieved directly from this public resource (Walley et al., 2016), and were scaled by color either individually by gene or absolute across all four genes of interest.

### Abiotic Elicitation of Maize Roots With CuSO_4_

Plant samples used for gene expression analysis were prepared previously (Mafu et al., 2018). Briefly, maize (var. Golden Queen) seed was germinated in the dark on wetted paper for 4 days at 23°C. Seedlings were transferred and grown hydroponically (Schmelz et al., 2001) for 12 days under 16/8 h light/darkness at 28°C, light intensity of 180 μmol photons m^-2^ s^-1^, and ∼60% relative humidity. A total of 1 mM CuSO_4_ or the corresponding water control was added to the hydroponic medium. Root samples were collected at the time points indicated, with three biological replicates per time point, and immediately frozen in liquid nitrogen for further analysis.

### Fungal Elicitation of Mature Maize Roots

Plants samples used for gene expression analysis were derived from a previous study (Mafu et al., 2018). Maize (var. Mo17) plants were grown in the greenhouse for 53 days in individual, 10 L pots and supplemented with 14-14-14 Scotts Miracle Grow fertilizer. Large nodal roots (≥2 mm dia) were punctured with a 0.6 mm dia steel pin at 1 cm intervals and inoculated with 10 μL of 1 × 10^7^ conidia mL^-1^ of *Fusarium verticillioides* (*F.v.*), *Fusarium graminearum* (*F.g.*), or water control at each wound site. To avoid damage to other tissues not undergoing treatment, sampling was limited to roots on the outer edge of the soil, in contact with the vertical plastic pot wall. Root samples were collected after 7 days and immediately frozen in liquid nitrogen before further processing.

### Quantitative Real-Time PCR (qPCR)

Gene expression analysis of *ZmCPS3* and *ZmCPS4* was performed on the CuSO_4_-treated or pathogen-treated root samples described above. Total RNA was isolated as described elsewhere (Kolosova et al., 2004) and cDNA was synthesized using SuperScript III First-Strand Synthesis Kit (Invitrogen) according to manufacturer’s instructions. Transcript abundance was measured using a BioRad C1000 Touch Thermo Cycler interfaced with a CFX96 Real-Time System, and iTaq Universal SYBR Green Supermix (BioRad) according to manufacturer’s protocols. Mean cycle threshold (Ct) values of at least two technical and three biological replicates were normalized using elongation factor *EF1α* as a validated reference gene (Schmelz et al., 2011), and fold change values were calculated using the 2^-ΔΔCt^ method. Gene-specific oligonucleotides used for qPCR analysis were *ZmCPS3* [forward: 5′-TGACGTGTGGATTGGGAAGG-3′, reverse: 5′-CGCTCTGTCTGGCTCAAAGA-3′], *ZmCPS4* [forward: 5′-CTCAGGCCAGCTTAACGAC-3′, reverse: 5′-CCTTGCCGATCCATACGTC-3′], and *EF1α* [forward: 5′-TGGGCCTACTGGTCTTACTACTGA-3′, reverse: 5′-ACA TACCCACGCTTCAGATCCT-3′].

### Sequence and Phylogenetic Analysis

Protein sequence alignments were generated using the CLCBio software package (Qiagen), followed by manual curation. Maximum likelihood phylogenetic analysis was performed using PhyML-aBayes version 3.0.1 beta (Guindon et al., 2010) with four rate substitution categories, LG substitution model, BIONJ starting tree, and 500 bootstrap repetitions.

## Results

### Active Site Determinants Suggest Distinct Functions of ZmCPS3 and ZmCPS4

**FIGURE 2:**
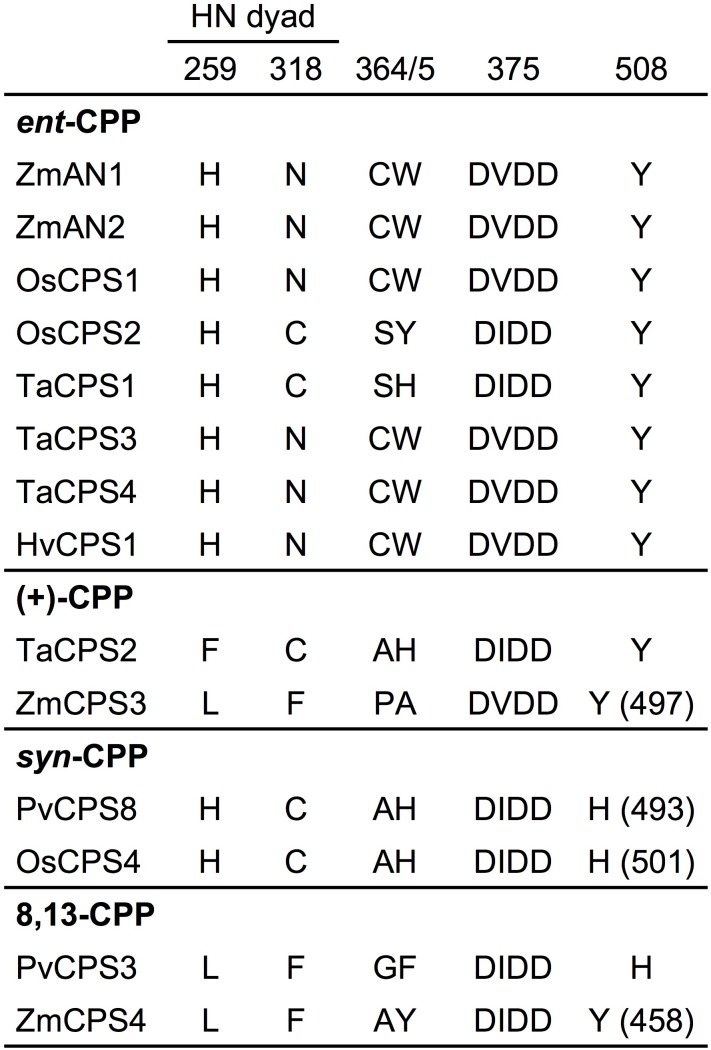
Active site determinants of monocot class II diterpene synthases. Illustrated is a protein sequence alignment highlighting key active site residues with demonstrated impact on product specificity of known monocot class II diTPSs. ZmCPS3 and ZmCPS4 feature distinct residues in select active site positions defining enzyme product specificity, thus suggesting a (+)-CPP synthase, 8,13-CPP synthase, or other specialized class II diTPS function for ZmCPS3 and ZmCPS4. Residue positions are numbered in reference to ZmAN2. Zm, *Zea mays*; Os, *Oryza sativa*; Ta, *Triticum aestivum*; Hv, *Hordeum vulgare*; Pv, *Panicum virgatum*.

The maize genome (B73 RefGen_v4) contains four class II diTPSs (Schmelz et al., 2014), which comprise the known *ent*-CPP synthases ZmAN1 and ZmAN2 located on chromosome 1 (Bensen et al., 1995; Harris et al., 2005), and the previously uncharacterized ZmCPS3 and ZmCPS4 positioned on chromosomes 4 and 10, respectively. ZmCPS3 and ZmCPS4 share a protein sequence identity of 55% with each other and 46–56% with ZmAN1 and ZmAN2. Phylogenetic analysis placed ZmCPS3 and ZmCPS4 separate from most *ent*-CPP synthases on a branch primarily consisting of class II diTPSs that produce prenyl diphosphates of specialized metabolism (Supplementary Figure S1). Although this suggested a role of both enzymes in specialized metabolism, the functional diversity of diTPSs in this group did not allow inference of possible ZmCPS3 and ZmCPS4 functions. Therefore, to inform biochemical analyses, we interrogated key active site residues with known impact on class II diTPS product specificity (Xu et al., 2007b; Mann et al., 2010; Jia et al., 2016, 2017; Mafu et al., 2016; Potter et al., 2016a; Pelot et al., 2018; Schulte et al., 2018). Previous studies identified a His-Asn catalytic dyad that is widely conserved among *ent*-CPP synthases, including ZmAN1 and ZmAN2, and was shown to direct class II diTPS catalysis toward *ent*-CPP formation (Potter et al., 2014, 2016b; Cui et al., 2015; Pelot et al., 2016). Neither ZmCPS3 nor ZmCPS4 possess a His-Asn catalytic dyad, but instead feature a Leu-Phe residue pair (Figure 2). These residues are consistent with a recently characterized 8,13-CPP synthase from switchgrass (*P. virgatum*; Pelot et al., 2018). In addition, presence of a Tyr residue in position 497 and 458 of ZmCPS3 and ZmCPS4, respectively, is consistent with known monocot *ent*-CPP and (+)-CPP synthases, but contrasts known *syn*-CPP synthases from rice and switchgrass that feature a His residue in this position (Potter et al., 2016a; Pelot et al., 2018). Although insufficient to allow an unambiguous functional annotation, these active site characteristics disfavored an *ent*-CPP or *syn*-CPP synthase activity, and supported a possible function of ZmCPS3 and ZmCPS4 as 8,13-CPP or (+)-CPP synthases or related specialized class II diTPSs.

### ZmCPS3 Functions as a (+)-CPP Synthase

**FIGURE 3:**
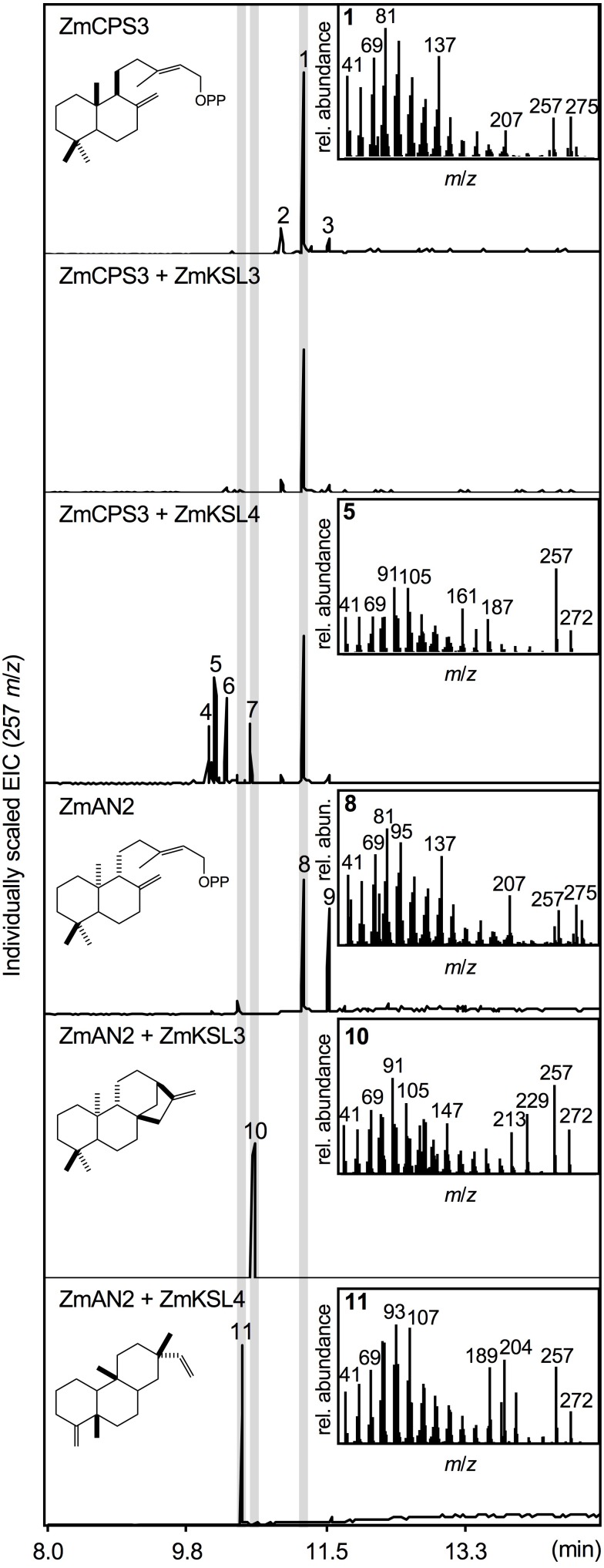
Functional characterization of the maize diterpene synthase ZmCPS3. Extracted ion chromatograms (EIC, *m*/*z* 257) and mass spectra of reaction products resulting from *E. coli* co-expression assays of ZmAN2 and ZmCPS3 alone, as well as in combination with the *ent*-kaurene synthase ZmKSL3 (Fu et al., 2016) or the dolabradiene synthase ZmKSL4 (Mafu et al., 2018). (+)-copalol (i.e., dephosphorylated copalyl diphosphate, CPP) **1**, geranylgeraniol (i.e., dephosphorylated geranylgeranyl diphosphate, GGPP) **2**, plasticizer contaminant **3**, pimara-8,14-diene **5**, *ent*-copalol (i.e., dephosphorylated *ent*-CPP) **8**, *ent*-CPP derivative **9**, *ent*-kaurene **10**, dolabradiene **11**, unidentified pimarane-type products **4**, **6**, and **7**. Where applicable, depicted structures represent the native prenyl diphosphate class II diTPS products, whereas spectra are derived from the corresponding dephosphorylated compounds that are formed during enzyme co-expression analyses by the activity of endogenous *E. coli* phosphatases.

To test the predicted enzyme activity of ZmCPS3, a synthetic full-length gene was co-expressed with a GGPP synthase from *A. grandis* using an *in vivo E. coli* expression platform engineered for diterpenoid production (Morrone et al., 2010). *In vivo* expression of class II diTPS enzymes using this system readily yields dephosphorylated products, presumably due to the activity of *E. coli* endogenous phosphatases and thus enables direct hexane extraction and analysis of the corresponding diterpene alcohols (Zerbe et al., 2013, 2015; Pelot et al., 2016; Mafu et al., 2018). For clarity, structures depicted below represent the native prenyl diphosphate products, but were detected via GC-MS and NMR analysis as the corresponding alcohols. Expression of ZmCPS3 resulted in a major product (compound **1**; Figure 3) with a retention time of 11.25 min and a fragmentation pattern showing dominant mass ions of *m*/*z* 137, 257, and 275 that closely matched the mass spectrum of copalol (i.e., dephosphorylated CPP) produced by ZmAN2 (compound **8**; Figure 3). Two additional byproducts detected in the ZmCPS3 product profile represented unconverted GGPP substrate (compound **2**) and an unidentified non-diterpenoid contaminant (compound **3**; Supplementary Figure S2).

Next we defined the stereochemistry of the ZmCPS3 product by co-expressing ZmCPS3 with characterized maize class I diTPSs that display catalytic specificity toward CPP substrates of different stereochemistries. ZmKSL3 converts *ent*-CPP into *ent*-kaurene (Fu et al., 2016), whereas ZmKSL4 forms dolabradiene from *ent*-CPP and pimarane-type diterpene olefins with (+)-CPP as a substrate (Mafu et al., 2018). As controls, we co-expressed ZmAN2 with either ZmKSL3 or ZmKSL4 to generate *ent*-kaurene (compound **10**) and dolabradiene (compound **11**), respectively (Figure 3 and Supplementary Figure S2). Co-expression of ZmCPS3 and ZmKSL3 did not result in any detectable class I diTPS product (Figure 3). The combined activity of ZmCPS3 with ZmKSL4 did not yield dolabradiene, but resulted in several pimarane-related products (compounds **4**–**7**), the most abundant of which was identified as pimara-8,14-diene (compound **5**) by comparison to reference mass spectra (Supplementary Figures S2, S3). These products are consistent with the previously reported activity of ZmKSL4 with the established (+)-CPP synthase, *A. grandis* abietadiene synthase variant D621A (Mafu et al., 2018). On the basis of these results, ZmCPS3 was designated as a (+)-CPP synthase.

### ZmCPS4 Produces 8,13-CPP and LPP

**FIGURE 4:**
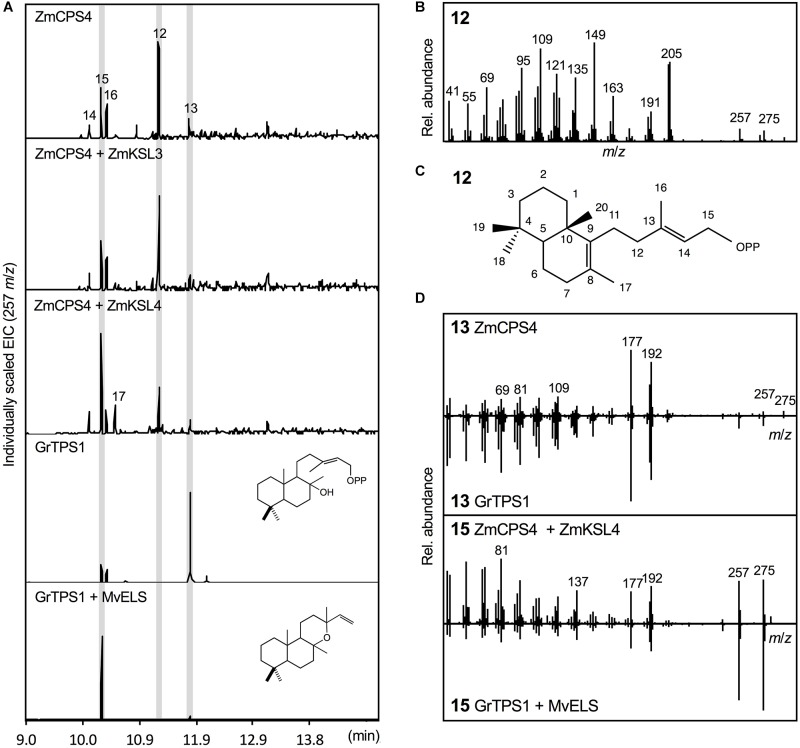
Functional characterization of the maize diterpene synthase ZmCPS4. **(A)** Extracted ion chromatograms (EIC, *m*/*z* 257) of reaction products resulting from *E. coli* co-expression assays of ZmCPS4 alone or in combination with the *ent*-kaurene synthase ZmKSL3 (Fu et al., 2016) or the dolabradiene synthase ZmKSL4 (Mafu et al., 2018). Expression of the LPP synthase GrTPS1 (Zerbe et al., 2013) was used to produce a labda-13-en-8-ol (i.e., dephosphorylated labda-13-en-8-ol diphosphate, LPP) standard **13**, and co-expression of GrTPS1 and the multi-functional class I diTPS MvELS (Zerbe et al., 2014) enabled the formation of a manoyl oxide **15** standard. **(B)** Mass spectrum of the ZmCPS4 product, 8,13-copalol (i.e., dephosphorylated 8,13-CPP) **12**. **(C)** Structural identification of the ZmCPS4 product as 8,13-CPP as verified by NMR analysis. Compounds **14**, **16**, and **17** represent unidentified LPP and 8,13-CPP derivatives. **(D)** Comparison of mass spectra of the ZmCPS4 products, compounds **13** and **15** to those of authentic standards of labda-13-en-8-ol (i.e., dephosphorylated labda-13-en-8-ol diphosphate, LPP) produced by GrTPS1 and manoyl oxide produced by combining GrTS1 and MvELS. Where applicable, depicted structures represent the native prenyl diphosphate class II diTPS products, whereas spectra are derived from the corresponding dephosphorylated compounds that are formed during enzyme co-expression analyses by the activity of endogenous *E. coli* phosphatases.

*Escherichia coli* co-expression of the full-length, synthetic gene encoding ZmCPS4 with the *A. grandis* GGPP synthase yielded a major product (compound **12**) with a retention time of 11.26 min, indicating a related but distinct compound as compared to (+)-CPP and *ent*-CPP formed by ZmCPS3 and ZmAN2 *in vitro*, respectively (Figure 4). Presence of signature mass ions of *m*/*z* 275 and 257 in the fragmentation pattern of this product indicated the expected labdane structure, but additional major mass ions of *m*/*z* 205 and 149 suggested a structure distinct from the common *ent*-CPP, (+)-CPP, or *syn*-CPP products observed in monocot crops (Xu et al., 2004; Harris et al., 2005; Wu et al., 2012). Indeed, the fragmentation pattern of compound **12** matched the product of a recently identified class II diTPS from switchgrass that forms 8,13-CPP (Supplementary Figure S2; Pelot et al., 2018). To further verify the identity of this ZmCPS4 product, nuclear magnetic resonance (NMR) spectroscopy was performed. For this purpose, large-scale *E. coli* co-expression cultures were used to produce an excess of 1 mg of the product, which was then purified using silica column chromatography and semi-preparative high-pressure liquid chromatography (HPLC). For the purified product, 1D ^1^H and ^13^C NMR spectra were acquired and validated the ZmCPS4 product as 8,13-CPP in comparison to published spectra (Supplementary Figure S4; Pelot et al., 2018).

In addition to the primary 8,13-CPP product, a minor, more polar, ZmCPS4 product with a retention time of 11.79 min was also observed (compound **13**; Figure 4). This product was identified as labda-13-en-8-ol diphosphate (LPP) based on comparison to the retention time and mass spectrum of LPP produced by a known LPP synthase from *G. robusta* (GrTPS1; Figure 4; Zerbe et al., 2013). Low abundance of this product prevented further stereochemical analysis via NMR. Additional byproducts (compounds **14**–**16**) present in the ZmCPS4 product profile represent manoyl oxide (compound **15**) as based on comparison to a manoyl oxide standard produced from the coupled reaction of *G. robusta* GrTPS1 and MvELS from *M. vulgare* (Zerbe et al., 2013; Pateraki et al., 2014), and a closely related unidentified diterpenoid (compound **14**; Figure 4 and Supplementary Figure S2). These products are likely resulting from rearrangement of the ZmCPS4 products 8,13-CPP and LPP after dephosphorylation by endogenous *E. coli* phosphatases, as has been described for various related class II diTPSs (Zerbe et al., 2015; Mafu et al., 2018; Pelot et al., 2018). To test for possible downstream products of 8,13-CPP and LPP, ZmCPS4 was co-expressed with the currently known maize class I diTPS functions, including the *ent*-kaurene synthase ZmKSL3 and the dolabradiene synthase ZmKSL4 (Fu et al., 2016; Mafu et al., 2018). No new products were detected when co-expressing ZmCPS4 and ZmKSL3 as compared to the expression of ZmCPS4 alone (Figure 4). When combining ZmCPS4 with ZmKSL4, compound **15** identified as manoyl oxide was significantly increased and, albeit at low abundance, an additional product (compound **17**) was formed that predictably represents a labdane diterpene olefin as based on characteristic mass fragments of *m*/*z* 272 and 257 (Supplementary Figure S2).

### ZmCPS3 and ZmCPS4 Show Distinct Patterns of Tissue-Specific Gene Expression

**FIGURE 5:**
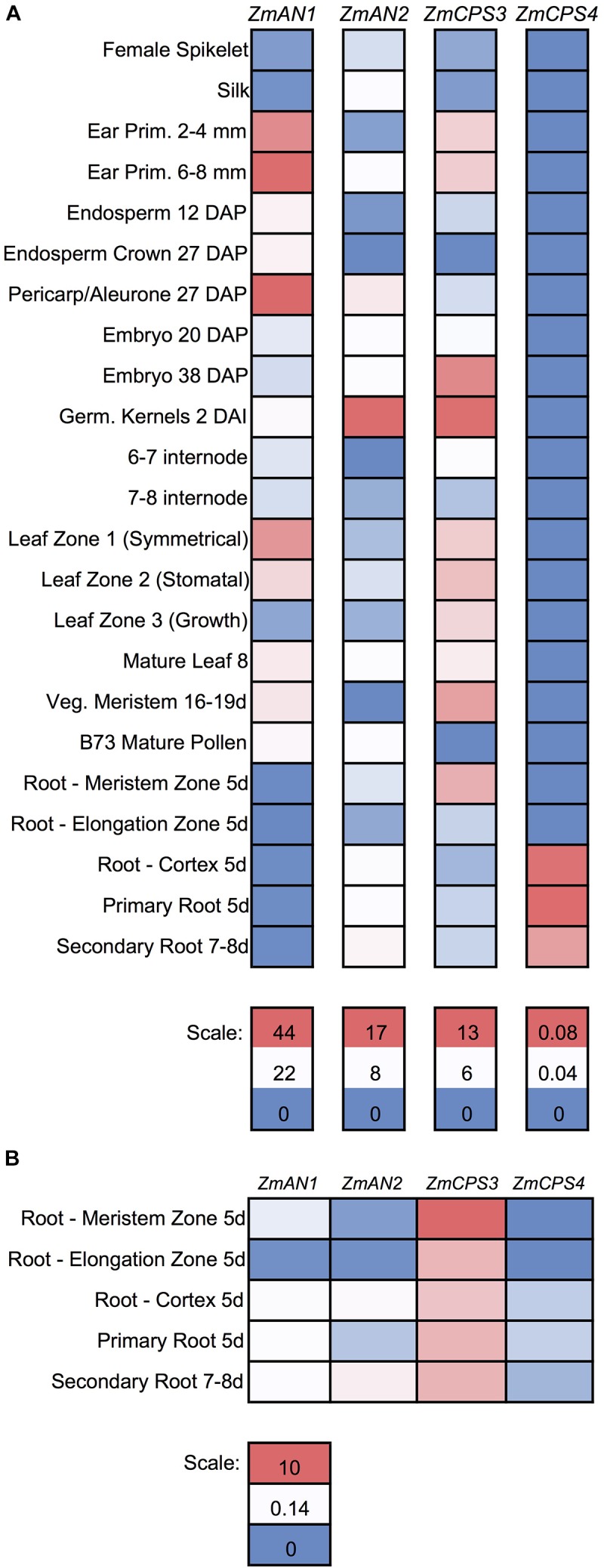
Gene expression of maize class II diterpene synthases across maize developmental stages and tissues. **(A)** Heat map showing the individually scaled mRNA expression (FPKM) of class II diTPS transcripts as based on a publically available transcriptome data panel comprising various maize tissue types and developmental stages (Walley et al., 2016). **(B)** Heat map showing only root samples, scaled to an absolute (not individual) scale. All samples are derived from maize genotype B73 with the exception of 5-days-old primary root (Mo17 inbred) and 2 cm tassels, 1–2 mm anthers, and mature pollen (W23 inbred).

To further investigate the functions of ZmCPS3 and ZmCPS4, we next examined their gene expression patterns using publicly available transcriptome and proteome data across various organs, tissues, and developmental stages of unstressed maize plants (Walley et al., 2016). With the exception of 5-days-old primary root (Mo17 inbred) and 2 cm tassels, 1–2 mm anthers, and mature pollen (W23 inbred), all samples were from the B73 genotype (Walley et al., 2016). Detectable transcripts of *ZmCPS3* were distributed across all organs and tissues with highest abundance included germinating kernels (Figure 5A). Notably, when specifically analyzing gene expression in roots, transcript of *ZmCPS3* was observed to be significantly more abundant with expression levels 5–481 times higher as compared to *ZmAN1*, *ZmAN2*, and *ZmCPS4* in the same tissue (Figure 5B). Transcript of *ZmCPS4* was detected at only trace levels and present exclusively in primary and secondary root and root cortex tissues (Figure 5). Consistent with observed transcript abundance, query of public proteome data (Walley et al., 2016) showed that protein levels of ZmCPS3 were highest in root tissues (Supplementary Figure S5). ZmCPS4 protein was not detected in the analyzed proteome.

### ZmCPS3 and ZmCPS4 Show Distinct Gene Expression Patterns in Response to Biotic and Abiotic Stress

**FIGURE 6:**
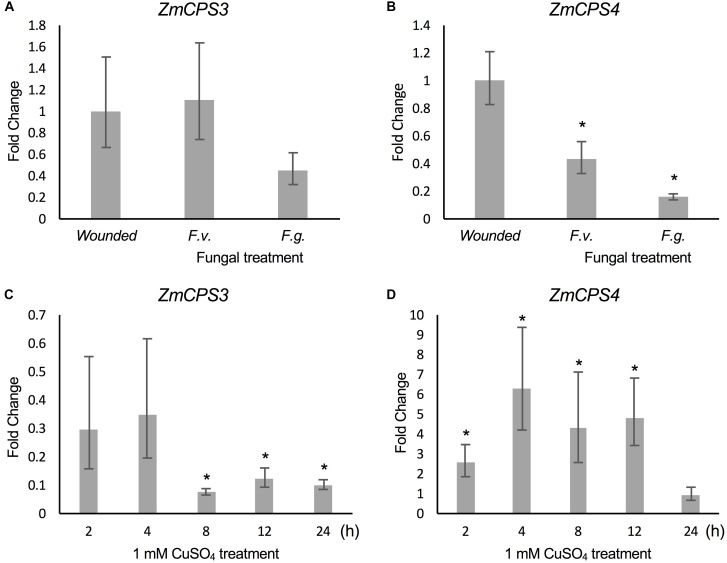
Transcript abundance of *ZmCPS3* and *ZmCPS4* under biotic and abiotic stress. Transcript abundance was measured by qPCR and is depicted as average fold change (2^-ΔΔCt^) of the transcript abundance of *ZmCPS3* **(A)** and *ZmCPS4* **(B)** in Mo17 roots incision-inoculated with live conidia, separately with *Fusarium verticillioides* (*F*.*v*.) and *F. graminearum* (*F*.*g*.). Plants treated by incision and application of water only were used as controls. Treatments occurred in 53-days-old plants with tissue harvests 7 days later (*n* = 4). Average fold change (2^-ΔΔCt^) of the transcript abundance of *ZmCPS3* **(C)** and *ZmCPS4* **(D)** in Golden Queen roots treated with 1 mM CuSO_4_ and sampled over 24 h as compared to a water-treated control (*n* = 3). A fold change of one is equal to no change from the control. Transcript abundance was normalized to the maize internal reference gene *EF1α.* Error bars represent propagated standard error of the mean fold change of the biological replicates. Asterisks indicate significant change compared to control with *P*-value < 0.05 as measured with two-tailed Student’s *t*-tests. A Dunnett’s test was used for fungal treated samples to compare to the single wounded control.

Previous studies demonstrated that *ZmAN2* gene expression is induced under both pathogen (*F.g.* and *F.v.*) and oxidative stress in above- (Harris et al., 2005; Christensen et al., 2018) and below-ground tissues (Mafu et al., 2018). In the context of these findings, the relatively higher transcript abundance of *ZmCPS3* in roots and the low but possibly root-specific expression of *ZmCPS4* suggested a role of these enzymes in belowground stress responses. To investigate this hypothesis, we analyzed transcript abundance of *ZmCPS3* and *ZmCPS4* using a previously reported set of samples of 53-days-old maize Mo17 roots incision-inoculated with fungal spores of *F.v.* and *F.g.* and harvested 7 days later (Mafu et al., 2018). Plants treated by incision-wounding only were used as controls. QPCR analysis showed no significant fold change in *ZmCPS3* transcript abundance in roots exposed to *F.v.* or *F.g.* as compared to wounded controls (Figure 6). On average, gene expression of *ZmCPS4* was significantly decreased in response to *F.v.* and *F.g.* elicitation as compared to control plants.

To examine *ZmCPS3* and *ZmCPS4* gene expression in response to abiotic stress, qPCR analyses were performed on two-week-old roots of maize (var. Golden Queen) plants that were exposed to 1 mM CuSO_4_ treatment (as a proxy for oxidative stress), previously shown to induce diterpenoid biosynthesis (Mafu et al., 2018). All treatments were compared to a water-treated control at each time point using the 2^-ΔΔCt^ method in which the control has a fold change of 1. Transcript levels of *ZmCPS3* in CuSO_4_-treated roots did not differ significantly from those observed in the roots of water-treated control plants at 2 and 4 h post treatment, but were significantly reduced at the 24 h post treatment time point (Figure 6) Conversely, *ZmCPS4* showed increased transcript abundance in CuSO_4_-treated roots as early as 2 h post treatment and with an up to sixfold change at 4 h, before decreasing again after 12 h, with a peak of sixfold increase in transcript abundance (Figure 6).

## Discussion

Rapidly increasing biotic and abiotic pressures can overcome the natural defense systems of plants, leading to substantial harvest losses in major food crops (Chakraborty and Newton, 2011; de Sassi and Tylianakis, 2012). Given the proven protective properties of crop-specific diterpenoid arsenals, a deeper knowledge of their biosynthesis and biological functions may aid new solutions to optimize crop stress resilience traits and mitigate yield loss (Schmelz et al., 2014). Elucidation of the enzyme activities of ZmCPS3 and ZmCPS4 completes the functional range of the maize class II diTPS family, which controls the early committed reactions responsible for the diversity of maize diterpenoid pathways.

Advances in the discovery and mechanistic analysis of diTPSs across the plant kingdom increasingly enable the prediction of diTPS functions (Zerbe and Bohlmann, 2015), as exemplified here for ZmCPS3 and ZmCPS4. However, accurate computational annotation of diTPS activities remains to be constrained by the vast sequence and functional space of the enzyme family, thus necessitating biochemical characterization. Modular co-expression assays with an expanding catalog of diTPSs of known substrate/product-specificity can be leveraged for efficient diTPS functional analysis, and were applied in this study for the identification of ZmCPS3 as a (+)-CPP synthase and ZmCPS4 as an 8,13-CPP synthase (Figures 3, 4). While absent in rice (Xu et al., 2007a), a (+)-CPP synthase has been identified in wheat, where (+)-CPP can be further converted by class I diTPSs to form labdane structures, such as pimara-8,15-diene, abietadiene, and isopimara-7,15-diene (Wu et al., 2012; Zhou et al., 2012). While corresponding end-products and physiological functions have yet to be discovered in maize and wheat, the wide distribution of (+)-CPP-derived diterpenoids in both angiosperm and gymnosperm species suggests roles in stress defense (Keeling and Bohlmann, 2006b; Zhou et al., 2012; Zerbe and Bohlmann, 2015). Similar to the recently demonstrated accumulation and predicted defensive functions of dolabralexins in maize roots (Mafu et al., 2018), a possible function of *ZmCPS3* in belowground stress responses could be hypothesized based on the higher transcript abundance in roots as compared to *ZmCPS4*, *ZmAN1*, and *ZmAN2*. However, the observed lack of elicited *ZmCPS3* expression in maize roots in response to *Fusarium* infection or oxidative stress did not support an inducible defense role for ZmCPS3-derived pathways and metabolites. Although *ZmCPS3* and its protein product were more highly expressed in roots as compared to other healthy tissues, its broad tissue- and development-wide presence may suggest a possibly more constitutive function. Tissue-wide expression levels of *ZmCPS3* suggest a conceptual parallel to the high constitutive levels of benzoxazinoid pathway enzymes and metabolites in maize seedlings, which have been shown to be important for the maize biotic and abiotic stress responses (Ahmad et al., 2011; Wouters et al., 2016). The largely constitutive and moderately inducible role is supported by a separate yet related study, where *ZmCPS3* transcript levels displayed an approximately twofold accumulation in the leaves of a resistant maize recombinant inbred line two weeks after challenge with gray leaf spot (*Cercospora zeina*; Meyer et al., 2017).

In addition to the (+)-CPP synthase ZmCPS3, characterization of ZmCPS4 as an 8,13-CPP synthase adds an uncommon scaffold to the diterpenoid network of maize (Bensen et al., 1995; Harris et al., 2005; Schmelz et al., 2014). Mechanistically, the proposed ZmCPS4-catalyzed reaction will proceed through terminal deprotonation of the common (+)-labda-13*E*-en-8-yl^+^ diphosphate intermediate at C-9 to yield 8,13-CPP, as opposed to the more typical deprotonation of the carbocation at the exocyclic C-17 methyl to form the (+)-CPP or *ent*-CPP isomers (Peters, 2010). ZmCPS4-catalyzed formation of LPP as a minor product will require hydroxylation at C-8 of the (+)-labda-13*E*-en-8-yl^+^ diphosphate intermediate (Falara et al., 2010; Caniard et al., 2012; Zerbe et al., 2012). Dual product activity is rarely observed in class II diTPSs (Caniard et al., 2012). Lacking available *ZmCPS4* maize mutants to enable *in planta* gene function studies, it can only be speculated if LPP represents a native ZmCPS4 product or results from a possibly reduced enzyme activity *in vitro* that could cause a slower conversion of the intermediary carbocation and thus facilitate water quenching toward formation of LPP. Notably, an 8,13-CPP synthase was also recently discovered in switchgrass, where the enzyme was characterized as a single product class II diTPS (Pelot et al., 2018). By contrast, an 8,13-CPP synthase function is absent in rice and wheat (Schmelz et al., 2014). Although a gene loss in these species cannot be excluded, this selective presence of 8,13-CPP synthases indicates the independent evolution of this function in maize and switchgrass. Similar to *ZmCPS3*, no pathogen-elicited gene expression was observed for *ZmCPS4*; in fact, transcript abundance was significantly decreased in response to *Fusarium* infection (Figure 6). This contrasts the well-established pathogen-elicited gene expression of *ZmAN2* (Harris et al., 2005; Schmelz et al., 2011; Christensen et al., 2018; Mafu et al., 2018), and may suggest that ZmAN2-derived pathways are the predominant inducible mediators of pathogen defense responses (Schmelz et al., 2011; Mafu et al., 2018). Conversely, CuSO_4_-induced expression of *ZmCPS4* in roots points to a possible role in belowground plant-environment interactions as also proposed for kauralexins and dolabralexins (Vaughan et al., 2015; Mafu et al., 2018) (Figure 6).

The identity of predominant pathway end-products derived from (+)-CPP, 8,13-CPP, and possibly LPP, as well as their corresponding roles in plant-environmental adaptation will require further investigation and ultimately the generation and analysis of defined genetic mutants in future studies. Currently, the biochemical characterization of ZmCPS3 and ZmCPS4 expands the known chemical landscape surrounding maize diterpenoid metabolism and completes the characterization of predicted class II diTPSs in the maize genome. Modular pathway networks composed of class II and class I diTPSs are likely operating in maize to convert the ZmCPS3 and/or ZmCPS4 products into a broader spectrum of specialized diterpenoids. *In vitro* formation of several labdane-type diterpenoids through the sequential activity of ZmKSL4, but not the *ent*-kaurene synthase ZmKSL3, with ZmCPS3 and ZmCPS4 support this hypothesis. However, no (+)-CPP, 8,13-CPP or LPP derivatives have yet been demonstrated in maize and the biological role of ZmCPS3/4 pathway branches has to be demonstrated *in planta*. Nevertheless, functional knowledge of all maize class II diTPSs will now enable a detailed investigation of modular diterpenoid-metabolic pathway branches in maize that are formed by class II diTPSs and known or thus far uncharacterized class I diTPSs as the biochemical foundation for maize diterpenoid diversity. Such insight can be of substantial value to elucidate and ultimately harness the genetic basis of crop stress resilience (Schmelz et al., 2014; Jez et al., 2016).

## Author Contributions

PZ conceived the original research and oversaw data analysis. KMM performed most experiments. L-TM performed sub-cloning experiments and assisted with enzyme characterization. YD and ES performed stress application to plants. KMM and PZ wrote the article with contributions from all authors.

## Conflict of Interest Statement

The authors declare that the research was conducted in the absence of any commercial or financial relationships that could be construed as a potential conflict of interest.

## Abbreviations

CPP: copalyl diphosphate
diTPS: diterpene synthase
GC-MS: gas chromatography mass spectrometry
GGPP: geranylgeranyl diphosphate
KSL: kaurene-synthase-like
P450: cytochrome P450.
