# Supplementary material for: Functional Characterization of Two Class II Diterpene Synthases Indicates Additional Specialized Diterpenoid Pathways in Maize (Zea mays)
Source: Front Plant Sci. 2018 Oct 23;9:1542. doi: 10.3389/fpls.2018.01542 (PMC6206430; doi:10.3389/fpls.2018.01542)
Supplement: Supplementary file 1 [file Data_Sheet_1.PDF]

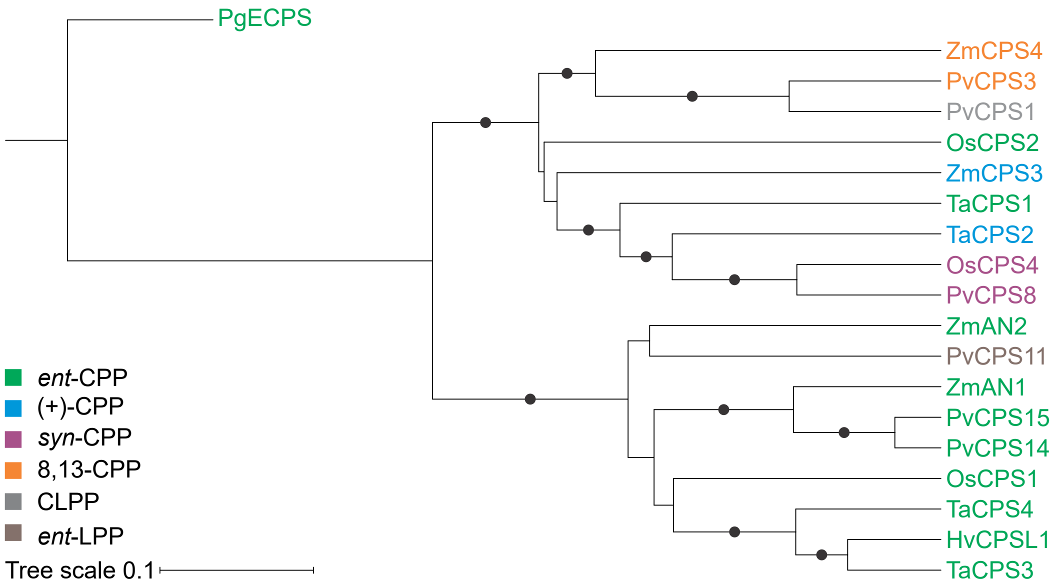

Supplementary Figure 1: Phylogenetic analysis of monocot class II diTPSs. Maximum Likelihood phylogenetic tree of biochemically characterized class II diTPSs from maize (*Zea mays*, Zm), rice (*Oryza sativa*, Os), wheat (*Triticum aestivum*, Ta), barley (*Hordeum vulgare*, Hv), switchgrass (*Panicum virgatum*, Pv). Tree rooted with the *ent*-CPP synthase from *Picea glauca* (Pg). Bootstrap values (500 repetitions) of 75% or higher are highlighted as black dots. Abbreviations: CPP, copalyl diphosphate; LPP, labdadienyl diphosphate; CLPP, clerodienyl diphosphate
