# Supplementary material for: Functional Characterization of Two Class II Diterpene Synthases Indicates Additional Specialized Diterpenoid Pathways in Maize (Zea mays)
Source: Front Plant Sci. 2018 Oct 23;9:1542. doi: 10.3389/fpls.2018.01542 (PMC6206430; doi:10.3389/fpls.2018.01542)
Supplement: Supplementary file 2 [file Data_Sheet_2.PDF]

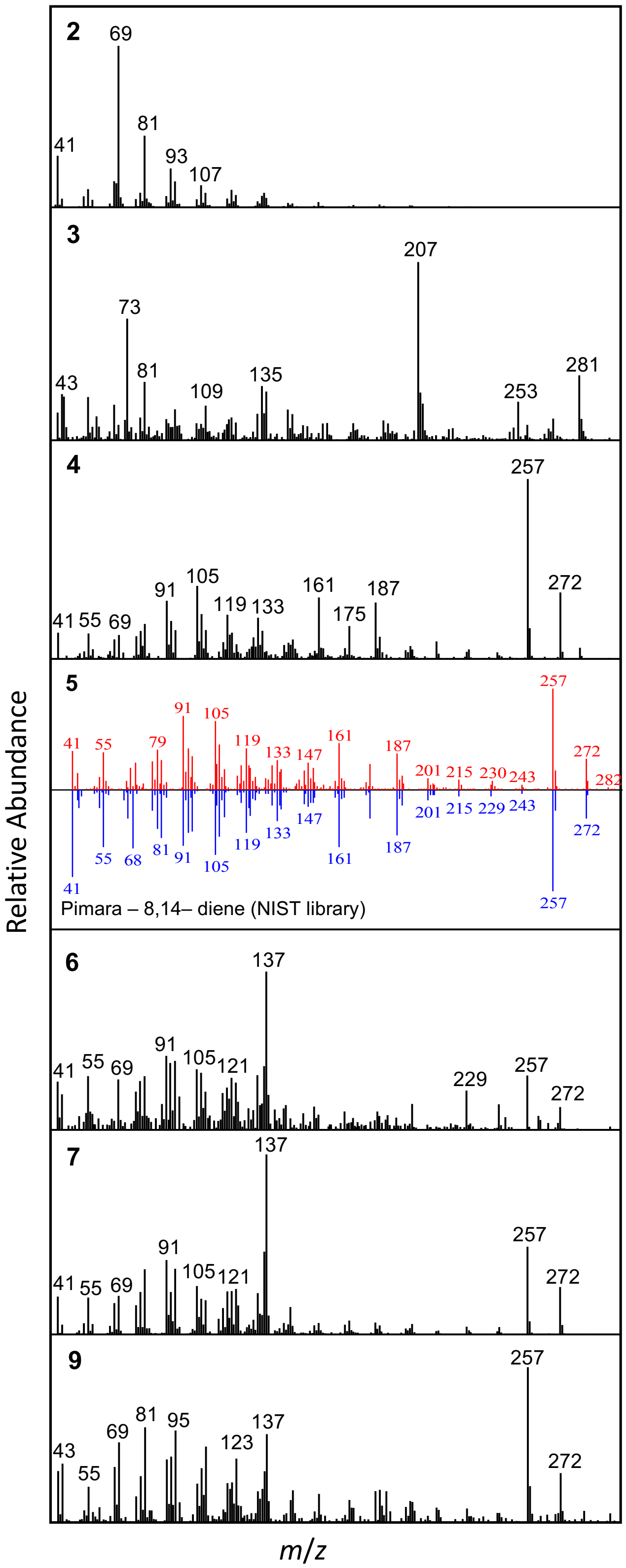

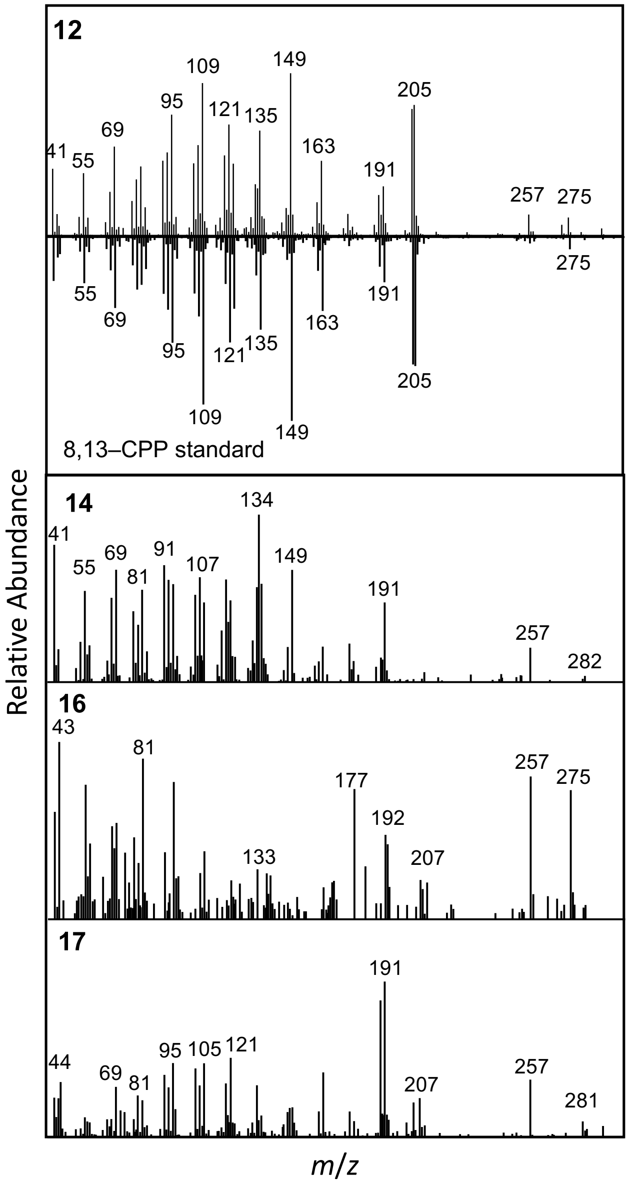

Supplementary Figure 2: Mass spectra of additional ZmCPS3 and ZmCPS4 co-expression products.

2 is the precursor GGPP. **3** is a plasticizer contaminant. **4** - **7** are ZmCPS3 + ZmKSL4 products. **5** (red) is compared to the National Institute of Standards and Technology (NIST) mass spectral library (Agilent) spectra of pimaradiene (blue). **9** is lower abundant ZmAN2 degradation product. **12** is 8,13-CPP compared to a standard made and purified from *E. coli* PvCPS1 (Pelot et al., 2018). **14** – **16** are byproducts of ZmCPS4. **17** is a ZmCPS4 + ZmKSL4 product. All identified structures are shown in Supplementary Figure 3.
