# Supplementary material for: Functional Characterization of Two Class II Diterpene Synthases Indicates Additional Specialized Diterpenoid Pathways in Maize (Zea mays)
Source: Front Plant Sci. 2018 Oct 23;9:1542. doi: 10.3389/fpls.2018.01542 (PMC6206430; doi:10.3389/fpls.2018.01542)
Supplement: Supplementary file 3 [file Data_Sheet_3.PDF]

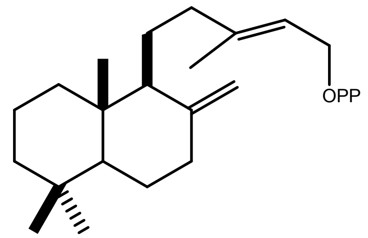

1. (+)-CPP

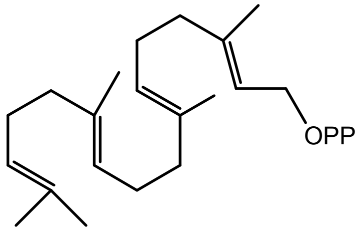

2. GGPP

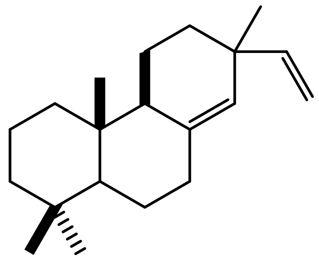

5. pimara-8,14-diene

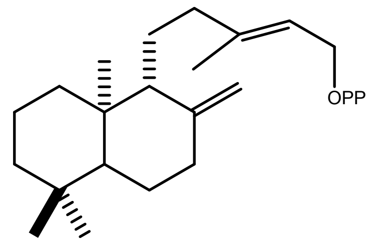

8. *ent*-CPP

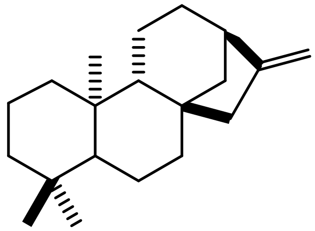

10. *ent*-kaurene

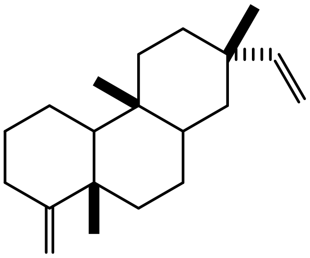

11. dolabradiene

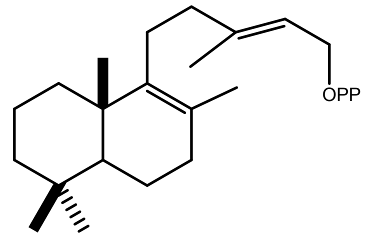

12. 8,13-CPP

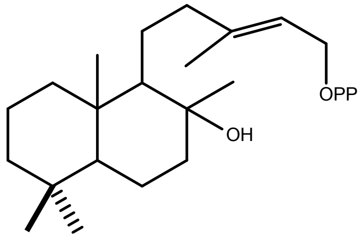

13. LPP

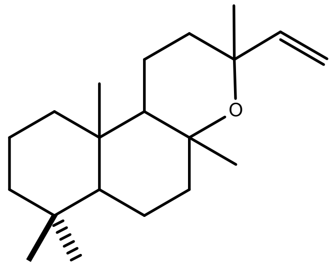

15. manoyl oxide

Supplementary Figure 3: Structures of compounds in this study. CPP = copalyl diphosphate; GGPP = geranyl geranyl diphosphate; LPP = labda-13-en-8-ol diphosphate.
