# Supplementary material for: Functional Characterization of Two Class II Diterpene Synthases Indicates Additional Specialized Diterpenoid Pathways in Maize (Zea mays)
Source: Front Plant Sci. 2018 Oct 23;9:1542. doi: 10.3389/fpls.2018.01542 (PMC6206430; doi:10.3389/fpls.2018.01542)
Supplement: Supplementary file 4 [file Data_Sheet_4.pdf]

Supplementary Figure 4: NMR of ZmCPS4 product, (+)-labda-8,13*E*-dien-15-ol (Compound **12**). Chemical shifts are comparable to the PvCPS1 product from Pelot et. al, 2018 (Pelot K. A., Chen R., Hagelthorn D. M., Young C. A., Addison J. B., Muchlinski A., Tholl D., Zerbe P. (2018). Functional diversity of diterpene synthases in the biofuel crop switchgrass. Plant Physiol. doi:10.1104/pp.18.00590). Previous research has shown that class II diTPS yields dephosphorylated products *in vivo* due to the activity of *E. coli* endogenous phosphatases, and thus the structure shown and analyzed here is the resulting alcohol (Zerbe et al., 2013; Zerbe et al., 2015; Pelot et al., 2016; Mafu et al., 2018).

^{13}C and ^{1}H NMR data (201 MHz and 800 MHz, respectively)

Position	δ_C (ppm) ZmCPS4	δ_C (ppm) PvCPS1	δ_H (ppm) ZmCPS4	δ_H (ppm) PvCPS1	J (Hz) ZmCPS4	J (Hz) PvCPS1
**1 a**	36.99	36.97	1.19 (m)	1.16 (m)		
**b**			1.84 (m)	1.82 (dt)		12.7, 2.3
**2 a**	19.09	19.07	1.51 (dt)	1.48 (dt)	12.8, 3.4	13.9, 3.6
**b**			1.62 (m)	1.59 (m)		
**3 a**	41.82	41.80	1.16 (m)	1.15 (m)		
**b**			1.44 (m)	1.41 (m)		
**4**	33.34	33.33				
**5**	51.92	51.90	1.14 (m)	1.12 (dd)		12.8, 2.0
**6 a**	19.08	19.07	1.42 (m)	1.40 (m)	12.9, 7.2	12.8,7.5
**b**			1.67 (dd)	1.64 (dd)		
**7 a**	33.66	33.64	1.96 (dd)	1.94 (dd)	17.5, 5.8	17.5, 6.4
**b**			2.06 (m)	2.04 (m)		
**8**	126.06	126.04				
**9**	140.16	140.13				
**10**	39.07	39.05				
**11 a**	26.79	26.77	2.01 (m)	1.98 (td)		11.8, 5.4
**b**			2.15 (m)	2.12 (td)		11.8, 4.9
**12**	40.18	40.16	2.08 (m)	2.05 (m)		
**13**	140.81	140.79				
**14**	122.63	122.60	5.46 (m)	5.43 (tq)		7.0, 1.4
**15**	59.46	59.44	4.19 (d)	4.16 (d)	6.9	7.0
**16**	16.38	16.36	1.74 (s)	1.71 (s)		
**17**	19.53	19.51	1.60 (m)	1.58 (s)		
**18**	33.33	33.32	0.91 (s)	0.88 (s)		
**19**	21.73	21.70	0.86 (s)	0.83 (s)		
**20**	20.11	20.09	0.97 (s)	0.94 (s)		

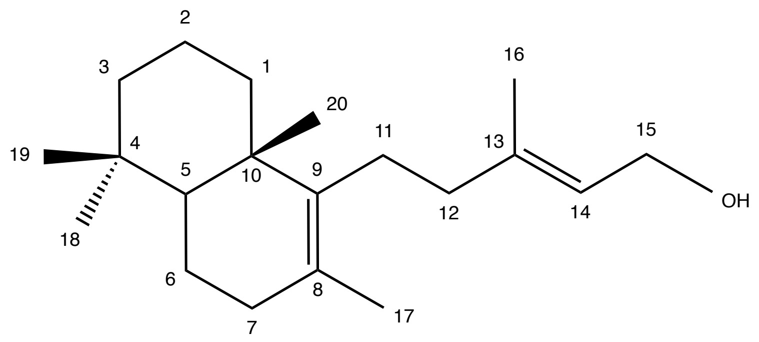
1
2
3
4
5
6
7
8
9
10
11
12
13
14
15
16
17
18
19
20
OH

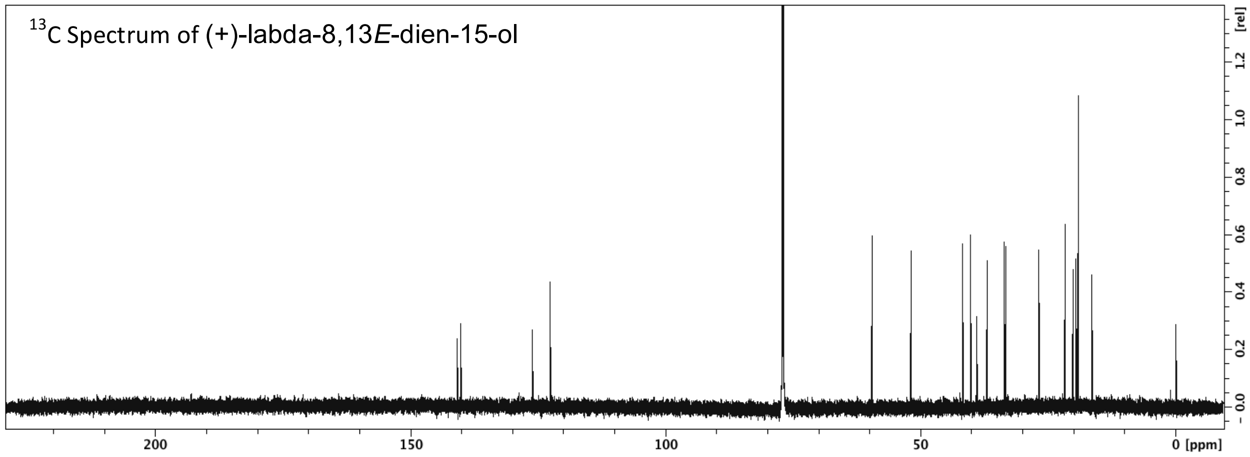
^{13}C Spectrum of (+)-labda-8,13*E*-dien-15-ol
200
150
100
50
0 [ppm]
[rel]

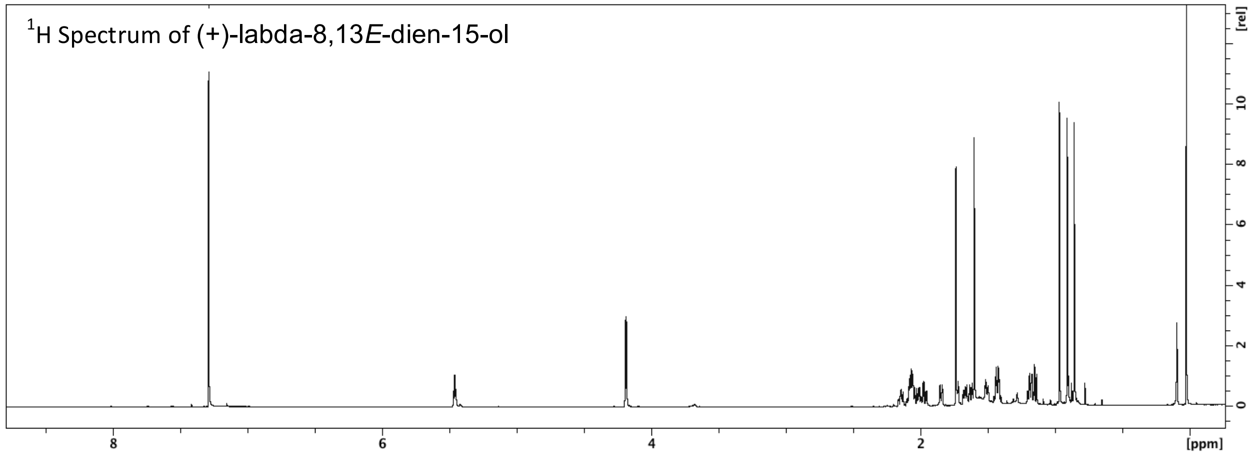
^{1}H Spectrum of (+)-labda-8,13*E*-dien-15-ol
8
6
4
2
[ppm]
[rel]
