# Supplementary material for: Functional Characterization of Two Class II Diterpene Synthases Indicates Additional Specialized Diterpenoid Pathways in Maize (Zea mays)
Source: Front Plant Sci. 2018 Oct 23;9:1542. doi: 10.3389/fpls.2018.01542 (PMC6206430; doi:10.3389/fpls.2018.01542)
Supplement: Supplementary file 5 [file Data_Sheet_5.PDF]

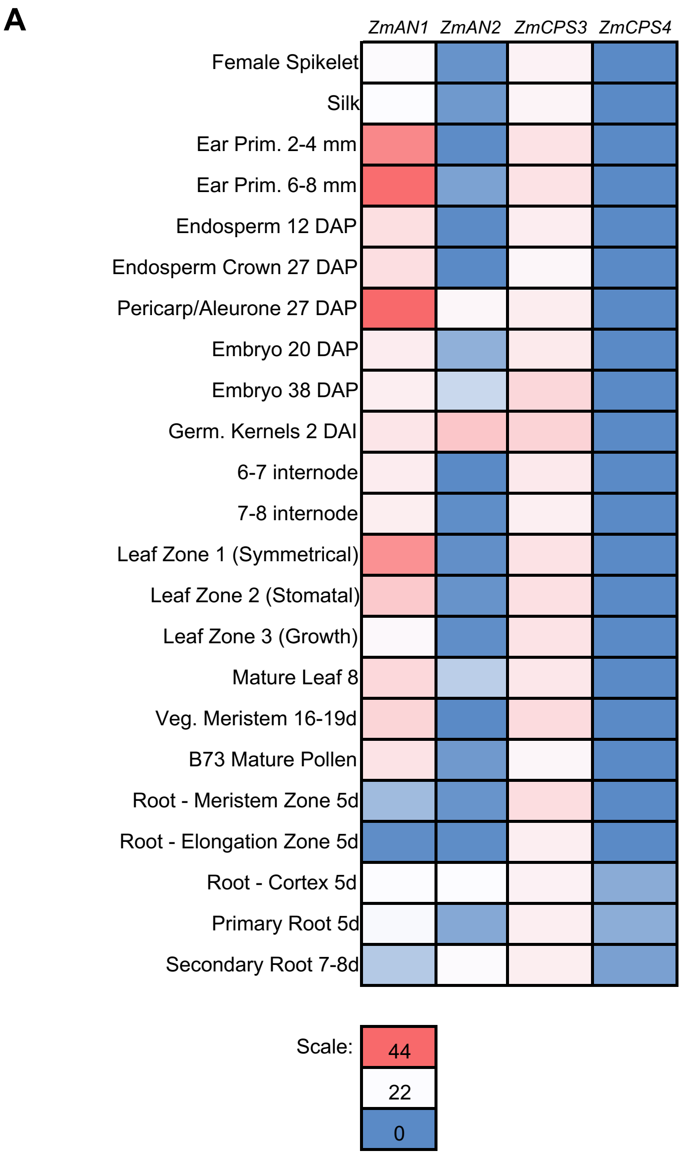

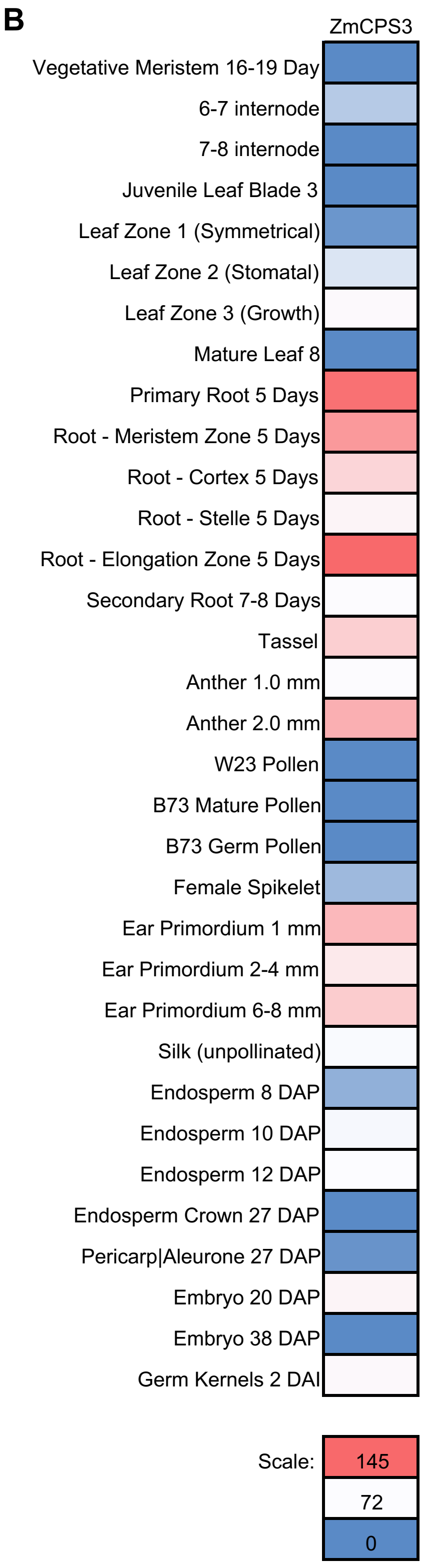

Supplementary Figure 5: Protein and transcript abundance of maize class II diTPSs.

(**A**) Transcript abundance of maize class II diTPS scaled relative to the highest and lowest abundance across all four gene transcripts. (**B**) Protein abundance of ZmCPS3. All data from Walley et. al, 2016.
