# Supplementary material for: Functional Characterization of Two Class II Diterpene Synthases Indicates Additional Specialized Diterpenoid Pathways in Maize (Zea mays)
Source: Front Plant Sci. 2018 Oct 23;9:1542. doi: 10.3389/fpls.2018.01542 (PMC6206430; doi:10.3389/fpls.2018.01542)
Supplement: Supplementary file 6 [file Data_Sheet_6.PDF]

Supplementary Table 1: Codon optimized sequences for *E. coli* used in this study.

Gene Name	Genebank ID	Maizegdb ID	Codon-optimized sequence
ZmCPS3	ZEAMMB73_Zm00001d024512	GRMZM2G068808	ATGGACTTGCCACGTCCGCAGGCACCGTGTTGTCGAAGACCGGCG<br>GATTTACGTCCGGCGCCATGTTGTATAGATGCGCCGCGCCCATCT<br>GATTTACGTGGTTGCGCCCCAGCTGCGGGCGCCCTTTGGAAGCCG<br>AGCGCCTCCTATCCGCGTGCCGCGGTCCCACCGGCGAGTGTTCGA<br>TTACATGACATCTCAGTGCGTGGCGAAGAAGACGAACAAAAAGAGA<br>TGTTACAACAGATGACCGATGGCGTTCGCGCAATGCTGGGTTCCAT<br>TGGTGACGGGGCCATCAACATTTCAGCCTATGATACGGCTTGGGTC<br>GCCTTGGTGAAATCACTGGAGGGCGGCAATGGTCCGCAATTTCCG<br>TCGTCGTTGCGTTGGATTGTTGAGAATCAATTACCGGATGGCAGCT<br>GGGGTGATGAAGAATTTTTTTTAGTATACGATCGCATGATTAACACT<br>TTGGCCTGCGTGATCGCACTGGAAAGTTGGGAAATCCACGCGGAC<br>ATGTGCGAGAAAGGTCTGTCCTTCATTCGTGAAAACTTATGGCGTT<br>TAGAACACAGCGGGTCCGATGATTGGATGGTGGTCAGCTTCGAAAT<br>CACCTTTCCGCAACTGCTGGAAATGGCCCGCGATCTGGGTTTAGAT<br>GTCCCGTGTGACGAACCTAGCCTGCGGGCCATTTATGCACGCCGT<br>GATGCTAAATTGGCGCGAATCCCGAAAGAGCTGCTCCATGCTAGTC<br>CGACTACCCTGCTTTTATCAATTGAAGGCATGCCGGGCTTGGACTG<br>GGAACGTCTGCTGAAACTGCAGTGCAGCGACGGTAGCTTCATGAG<br>CTCGCCGGCACCGACTGCTTACGCCCTGATGCAAACAGGCGATAC<br>AAAATGCTTAGAATTTCTCAATGGCATCGTAAGCAAATTTTCAGGTG<br>GTGTCCCTTTTACGTATCCAGTCGATCTGTTCGAGCACCTGTGGGT<br>TGTGGACCGCATTGAACGTCTGGGTATCGGTCGCCATTTCACCGG<br>CGAAATTAAAGAATGTCTGGAATACGTGCACCGCTATTGGGGCGAC<br>GAGGGTCTGCCAGCAACCCGTGATGGCCCGGTGAGCGATGTGGAT<br>GATACCGCAATGGGTTTCCGTCTGCTGCGCCTGCACGGTTATGAC<br>GTGAGTCCCTCTGTTTTCAAGCATTTTGAACAAGATGGTGCATTCTA<br>CTGCTATCCTGGTCAGTCCAATAAGAGCGTGACCGCCATGTATAAC<br>CTCTATCGGGCGTCCCAAGTCGCGTTTCCTGGTGAGGACGAGCTG<br>CGCCGTGCCGAAGCATACTCTCGTGAATTTCTGTGTCGTCGCCGT<br>GCTTCTGGTGAACTGAAGGATAAATGGGTTATTCCCAAAGATCTGC<br>CGGGCGAGGTAGCGTACGCCCTGGACGTTCCCTGGAAAGCAAGC<br>CTCCCCCGCATTGAAACCCGAATGTATCTGGAGCAATACGGTGGC<br>GCAGACGACGTGTGGATTGGGAAGGTGCTGTACCGCATGAGCTTG<br>GTGAACAATGAACTGCTGCTGCGCACAGCGCAGGCAGACTTTCGC<br>TCGTTTCAGCGTCAGTGCAAACTGGAATGGCATGGCTTAAGAAAAT<br>GGGCGTCCCGCCGCAATTTACAGGCTTACGGGGTCACGTCAAATT<br>CGACCCTGCGATCTTATTTTCTGGCGGCGGCCAGCATTTTCGAACC<br>GGATCGTGCGACGGAGCGCCTGGGTTGGGCGCGCACGGCGGTTC<br>TGGCTGAGGCGGTAAGCAGCTGCCTGCGCGATGGCCGTTGCTCAC<br>GCGCCGATGGAATGCTGCGCGAATTGACGAGCGGCATTCACTTAA<br>GAAATGATGATAATCCGGCGGCATCACTGGTTCATGCGCTGCATGA<br>GTTAATAGGCCTTCTTGCGTTCGATAACGCGTCTTATAATTCTCTGC<br>TGGACGCTTGGAAACAATGGCTGGCCATGTGGACCGCGCAGGGTC<br>ACGAGGGCTCCGTTGCGCTGCTGCTTGTCCGCACTGTGGAGATTT<br>GTAGTGGCCGTCGTCGAAGCGCCAGTGCAACCGACGATGGACAG<br>GCCCATTCACTGAGCGAATATAGTCAGCTGGAACAGCTGACTAGCA<br>GTGTGTGCAGTAAACTGGCCGCGGCAGGTACAGGTGATGGCGATG<br>ATCTGAGCCCAGCAAGCGTTGAAGATGCGGCCGATCGTCATCGTG<br>TTGATCTGGAAATGCAGACCTTGGCACGCTGCGTTCTCCGCAGCC<br>GCAGTAGTATAGACGCGGTAACCCGGCAAACCTTTCTGCACGTGG<br>CACGTTCCTTTTACTACGTGGCGCACTGTTCCGCGCGCACCGTGG<br>ACGCCCATATTTCTAAAGTTCTGTTTGAAGATGTTGTGTAA

Supplementary Table 1: Constructs used in this study.

Gene Name	Genebank ID	Maizegdb ID	Codon-optimized sequence
ZmCPS4	ZEAMMB73_Zm00001d048874	AC218998.2_FG011	ATGGGTGCCACAAAGGGTGAAAAACTAAAAACACTCGGTCATGATT<br>TACTTGTTCCTGCCACTCCTCAACACAAGGATAAACAAAGTGGCAT<br>GCCCGAAATGATTGAGGCCATCCGAGTAGCCCTTCGTTCAATGGG<br>AGACGGTGAGATTAGCATTTCAGCATATGACACGGCATGGGTTGCA<br>CTTGTAAGGAGTTTGAATAATAATGGAGATGATGGACCAGAGTTCC<br>CATCTTGCATTGATTGGATTGCTCAAAACCAGCTTCCCGATGGATC<br>ATGGGGTCATGACATCTTCTTCCTAGCCCAAGATCGGATCATCAAC<br>ACCCTAGCCTGCGTCATCGCATTGAAATCATGGAAAATCCACGATG<br>ATGCGTGCACAAAAGGTCTATCATTCATCACTGAAAATATGTGGAG<br>GTTGACCAGGGATGATGAGAATTGGGCACTATCAGGCTTCGAGATT<br>ATATTCCCTATGTTGCTAGAGAAGGCCAAACACCTAGGCATCGACA<br>TACCTTTGGATGATCCCATGTTGGAAGCTATACGAGCCAAAAGAGA<br>ACTCAAGTTAAACAAGATCCCAAGAGAAGCACTTCATGCTGAACCA<br>ACAACTTTCCTTCTAAGCATAGAAGGGATGCCAGGTTTGGACTGGA<br>AAAGGCTACGTAAGCTCCAGTGTCCAGATGGCTCCTACATGTCTTC<br>GCCTGCTCCCACAGCTTATGCTCTAATGCAGACTGGGGATGCCAA<br>GTGTTTTGAGTTCCTTGATAAACTGATCGACAAGTTTAACGGAGGA<br>GTACCTTTTGTTTACCCAATGGAGATGTTTGGGCGCTTATGGGCTG<br>TGGACCGGTTGGAGAGGCTGGGCATATCAGGTTATTTCAAGAGCG<br>AAATTGAGGACTACTTAGATTATGTTTACAGGCACTGGAGTGAGGA<br>AGGGTTGGCCTATACGAAGGGCTGCCTGGTGAAGGACATCGATGA<br>CACGGCCATGGGTTTCCGCCTCCTACGACTGCACGGCTACGACCA<br>TGTCTCTCCTTGTGTTTTCAAGCGGTTCGAGAATGGCGACGGGCAG<br>TTCGTGTGCTACGCAAGGCAATCGAGCCAGTCGGTAAGCGCCATG<br>TACAACCTGTACCGAGCTGCTGACCAGGCCTCGTTCCCCGGTGAC<br>GACGACGACCATGTCCTCCGGCGCGCCAGGAGCTACAGCCGTGC<br>GTTCCTCCGGCAGAGACGAGCCTCAGGCCAGCTTAACGACAAGTG<br>GATCATCTCCGAGGGCTTGCCCGACGAGGTTGGCTATGGTCTGGA<br>TTTCCCTTGGGGAGCAAGCCTGCCACGTATTGAGACGAGAATGTAT<br>CTTGAGCAATACGGTGGAAGTCGTGACGTATGGATCGGCAAGGTT<br>CTCTACAGAATGAACATGGTGAGCAACGACATGTACCTCGAGGTGG<br>CGAAAGTCGATTTCAGCAACTTTCAGAGACTATGCCGACTAGAGTG<br>GCACGACCTCAAAAGGTGGTGTGACAAGAGCGACCTTGAAACGTA<br>CGGCGTGGCTCCGGGCGGCGCGCTGAGAGCCTACTTCCTGGCGG<br>CAGCCTGCATCTTCGAGCCGGGCCAAGCAGCAGAGCGCCTGGCTT<br>GGGCACGCGCCGCGGTGCTCGCCAAGGCCATCTCGTGCTGCTTG<br>TTGAGCAACAACGACACATGCGCTTGTAATAAGACGACGGCCGAAT<br>GGCTCGTCCGGGAATTCACCAATGGTGACAATGTTGCTGGCTACTA<br>CTACGACTACAACCCAGCAAGGAGAGACGACGACGAGCCGAATTC<br>ACCAGCATGGGGGGCCAGCAGCCTCGCCGGTGTTCTTCGTGAGCT<br>CGTCCACTTGCAGGCGTCCGGGAATGCTGCTGTCGCCGAATGTCT<br>TCGTGGAGCTTGGATGGAATGGCTCATGGCATGGACTGAAAAGGA<br>GACGGAAGAAGCGTCACACGCAGGAGATACAGCGCTGCTGCTAGC<br>TCGCACAGTCGAGATATGCTCAGGAAGGCTCCGTGGTACGGAACA<br>GGATCTCGAGCTACGTCTGCCGGATTACTCCAAGCTCCAGCAGCT<br>CACTAGATGCATATGCTCCAGACTAGCCACTGAAGCTCCTGCTCTG<br>ATCGAATCAAACGTCCTCAAACAGAATGGAGAAACCATGGACAAGG<br>TTGATGCCCTGGACAGGATGGTTGGACTCGAGATGAGAGAACTGG<br>CTCAATGTGTTTTCCGCAGCGGCGGCAGCTCCGTGGACAGAGAGA<br>CGAGGCAGACGTTTCTCCACGTGACCAAGAGTTACTACTACGTTGC<br>ACTCTGCTCGCCGGAAACACTTGAGCATCACATCTCCAAAGTCTTG<br>TTTGAGGATGTCGTCTAGAGCTCGGCGCGCCTGCAGGTCGACA
